# The effect of sedation and/or analgesia as rescue treatment during noninvasive positive pressure ventilation in the patients with Interface intolerance after Extubation

**DOI:** 10.1186/s12890-017-0469-4

**Published:** 2017-09-15

**Authors:** Yue-Nan Ni, Ting Wang, He Yu, Bin-Miao Liang, Zong-An Liang

**Affiliations:** 1 Department of Respiratory and Critical Care, No.37 Guoxue Alley, Chengdu, 610041 China; 2 Department of Critical Care Medicine, West China School of Medicine and West China Hospital, Sichuan University, Chengdu, 610041 China

**Keywords:** Noninvasive positive pressure ventilation, Interface intolerance, Analgesia, Mortality

## Abstract

**Background:**

Sedation and/or analgesia can relieve the patient-ventilator asynchrony. However, whether sedation and/or analgesia can benefit the clinical outcome of the patients with interface intolerance is still unclear.

**Methods:**

A retrospective study was performed on patients with interface intolerance who received noninvasive positive pressure ventilation (NIPPV) after extubation in seven intensive care units (ICU) of West China Hospital, Sichuan University. The primary outcome was rate of NIPPV failure (defined as need for reintubation and mechanical ventilation); Secondary outcomes were hospital mortality rate and length of ICU stay after extubation.

**Results:**

A total of 80 patients with oral-nasal mask (90%) and facial mask (10%) were included in the analysis. 41 out of 80 patients received sedation and/or analgesia treatment (17 used analgesia, 11 used sedation and 13 used both) at some time during NIPPV. They showed a decrease of NIPPV failure rate, (15% vs. 38%, *P* = 0.015; adjusted odd ratio [OR] 0.29, 95% confidence interval [CI] 0.10–0.86, *P* = 0.025), mortality rate (7% vs. 33%, *P* = 0.004; adjusted OR 0.14, 95% CI 0.03–0.60, *P* = 0.008), and the length of ICU stay after extubation.

**Conclusion:**

This clinical study suggests that sedation and/or analgesia treatment can decrease the rate of NIPPV failure, hospital mortality rate and ICU LOS in patients with interface intolerance after extubution during NIPPV.

**Electronic supplementary material:**

The online version of this article (10.1186/s12890-017-0469-4) contains supplementary material, which is available to authorized users.

## Background

The early use of noninvasive positive pressure ventilation (NIPPV) can reduce the reintubation rates of patients after extubation for its role in decreasing the work of breathing and improving gas exchange [1–7]. However, the interface intolerance could result in patient-ventilator asynchrony [8, 9], which could also cause the discontinuation of NIPPV and thus lead to unplanned requirement for endotracheal intubation with a rate up to 9–22% [10].

Sedation and/or analgesia can release the discomfort of patients about NIPPV interface [11, 12]. Whether sedation and/or analgesia during NIPPV is safe and feasible has been assessed in several clinical trials but the results still remains unclear. In the review of Hilbert, the author held the view that although lack of evidence, the mask intolerance could be the ideal indication of sedation and/or analgesia and is clearly to avoid intubation [13]. On the contrary, Conti thought patients could not benefit from sedation and/or analgesia [14].

Therefore, the role of sedation and/or analgesia in reducing the rate of NIPPV failure in post extubation patients with interface intolerance is unclear.

Thus, we hypothesized that the use of sedation and/or analgesia could decrease the failure of NIPPV and a retrospective study was conducted to examine the effect of sedation and/or analgesia on post-extubation patients with interface intolerance.

## Methods

### Study design

Patients hospitalized between December 2014 to August 2016 at 7 intensive care units (ICUs) in West China Hospital, Sichuan University, China were studied in the clinical investigation. The study was approved by the Institutional Ethical Committee for Clinical and Biomedical Research of West China Hospital (Sichuan, China). Written informed consent was waived given that the investigation was a retrospective observational study and involved no therapeutic intervention.

All adult patients received NIPPV after extubation were screened (the weaning protocol could be seen in Additional file 1 and the inclusion criteria for patients used NIPPV directly after extubation could be seen in Additional file 2). Patients were eligible if they were recorded as interface intolerance (claimed by patients themselves) in the medical records and/or nursing records and received more than 2 h of NIPPV after extubation, or fulfiled at least one of the following criteria for using NIPPV directly after extubation: 1) Age older than 65 years; 2) Heart failure as the primary indication for mechanical ventilation; 3) Moderate to severe chronic obstructive pulmonary disease; 4) An Acute Physiology and Chronic Health Evaluation II(APACHE II) score higher than 12 on extubation day; 5) Body mass index of more than 30 (calculated as weight in kilograms divided by height in meters squared); 6) Airway patency problems, including high risk of developing laryngeal edema; 7) Inability to deal with respiratory secretions (inadequate cough reflex or suctioning > 2 times within 8 h before extubation); 8) Difficult or prolonged weaning, in brief, a patient failing the first attempt at disconnection from mechanical ventilation; 9) 2 or more comorbidities; 10) Mechanical ventilation for more than 7 days.

Patients with short duration of NIPPV were excluded to guard against reverse causality because these groups of patients were already discontinued from the NIPPV when sedation and/or analgesia plays a role.

### Data extraction

We collected information on each patient’s age, gender, illness severity obtained at ICU admission and extubation manifested as the score of Acute Physiology And Chronic Health Evaluation (APACHEII), arterial blood gases prior to NIPPV started (half an hour before extubation), interface used to apply the NIPPV. After NIPPV treatment started, we recorded the following variables: interface for NIPPV, the history of NIPPV usage before mechanical ventilation, the maximum inspiratory positive airway pressure and expiratory positive airway pressure, respiratory rate, inspired fraction of oxygen, body temperature, heart rate, arterial blood gases and level of consciousness–sedation by Richmond Agitation Sedation Scale (RASS). At each change of ventilator settings, we registered the same variables and the administration of sedatives (dexmedetomidine, propofol) or analgesia (fentanyl, sufentanyl). In addition, the arterial blood gas was recorded at the beginning of the sedation and/or analgesia treatment and 4 h after the treatment began. During the study, all patients would be monitored until they were discharged from the hospital or dead. We obtained data of the rate of NIPPV failure, hospital mortality, length of ICU stay (ICU LOS) and rate of delirium after extubation. The failure of NIPPV was defined as the requirement for intubation and invasive mechanical ventilation (the standard of reintubation could be seen in Additional file 1).

### Outcomes

The outcomes included the failure of NIPPV, the hospital mortality and ICU LOS after extubation.

### Statistical analysis

Consecutive variables were reported as mean ± standard deviation or median (Inter-Quartile Range [IQR]), while categorical variables were reported as frequency and proportion. The student’s t, Mann-Whitney U-test and Kruskal-Wallis tests were used for comparisons between continuous variables and the Chi-squared test or Fisher’s exact test were for comparisons between categorical variables.

To assess the relationship between the use of sedation and/or analgesia with the rate of NIPPV failure and the hospital mortality, we structured a logistic regression model and the OR was adjusted by variables independently associated with failure of NIPPV or hospital mortality that had *P* value less than 0.10. Kaplan-Meier curves were analysed to assess the time from extubation to failure of NIPPV or death. In addition, the time was compared by means of the log-rank test.

All the analyses were performed with SPSS 19.0 (SPSS Inc) and 2-sided *P*-value less than 0.05 was considered statistically significant.

## Results

### Patients

**Fig. 1:**
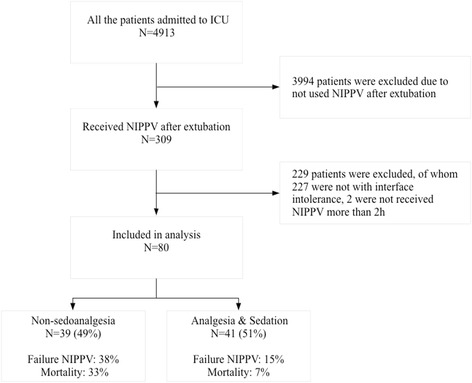
Flowchart of the study NIPPV, noninvasive positive pressure ventilation; ICU, intensive care unit

From December 2014 through August 2016, a total of 4913 patients were admitted to the 7 ICUs. Among them, 309 patients received NIPPV directly after extubation and 80 patients were recorded as interface intolerance in the medical records and/or nursing records (Fig. 1). Forty-one out of 80 patients (51%) received intravenous sedation and/or analgesia at any time during NIPPV: 17 used analgesia, 11 used sedation and 13 used both. The sedation drugs included propofol and dexmedetomidine, while the analgesia drugs included fentanyl and sufentanil. And 90% of the patients used oral-nasal mask while the other 10% used facial mask.

### Characteristics at inclusion

The characterisitics of the patients at enrollment were shown in the Table 1 **.** The mean age and proportion of male patients in sedation-analgesia and non-sedoanalgesia groups were 68 vs.78 (*P* = 0.077) and 73% vs. 64% (*P* = 0.471), respectively. There was no significant difference in the score of APACHE II at ICU admission (17.95 ± 6.87 vs. 19.28 ± 6.49, *P* = 0.376) nor at the extubation (10.61 ± 3.89 vs. 10.97 ± 5.29, *P* = 0.725) as well as PaO_2_/FiO_2_ before extubation (230.43 vs. 260.59, *oP* = 0.059) between the two groups. We did not find significant difference in body temperature, respiratory rate, heart rate, blood pressure as well as the pH and PaCO_2_ before extubation.

**Table 1:** Baseline characteristics of patients according to sedonalgesia status during NIPPV

	Sedation and/or analgesia (*N* = 41)	Non sedoanalgesia (*N* = 39)	*P*
Age^c^, years	68(54 ~ 78)	78(62 ~ 83)	0.077
Male^b^	30 (73%)	25 (64%)	0.471
APACHE II at ICU admission^a^, points	17.95 ± 6.87	19.28 ± 6.49	0.376
APACHEII at extubation^a^, points	10.61 ± 3.89	10.97 ± 5.29	0.725
NIPPV before mechanical ventilation^b^	11 (27%)	11(28%)	0.890
Diagnosis			
Pneumonia^b^	24(59%)	25(64%)	0.610
AECOPD^b^	13(32%)	12(31%)	0.928
Other^b^	4(10%)	2(5%)	0.676
Interface			0.476
Oral-nasal mask^b^	38(93%)	34(87%)	
Nasal mask^b^	3(7%)	5(13%)	
Before extubation			
OI^a^	230.43 ± 62.33	260.59 ± 77.83	0.059
pH^a^	7.44 ± 0.03	7.44 ± 0.04	0.891
PaCO_2_^a^, mmHg	50.46 ± 10.26	47.27 ± 11.43	0.193
body temperature^a^	36.73 ± 0.38	36.91 ± 0.53	0.090
RR^a^, bpm	20.61 ± 4.69	22.59 ± 6.24	0.112
HR^a^, bpm	105.59 ± 15.78	101.36 ± 17.72	0.263
SBP^a^, mmHg	140.34 ± 22.69	136.92 ± 18.93	0.463
DBP^a^, mmHg	74.51 ± 13.16	73.41 ± 10.53	0.680
Half an hour after extubation			
OI^a^	266.94 ± 325.79	225.47 ± 67.65	0.438
pH^a^	7.44 ± 0.05	7.43 ± 0.04	0.293
PaCO_2_^a^, mmHg	50.34 ± 11.97	50.71 ± 13.53	0.897
RASS at start of NIPPV			
-2 to −1 points^b^	15(37%)	3(8%)	0.003
0 to 2 points^b^	26(73%)	36(72%)	
IPAPmax^a^, cmH_2_O	13.00 ± 2.58	12.74 ± 2.28	0.639
EPAPmax^a^, cmH_2_O	5.56 ± 0.95	5.44 ± 1.17	0.599

*AECOPD* acute exacerbation of chronic obstructive pulmonary disease, *APACHE II* The Acute Physiologic and Chronic Health Evaluation, *DBP* diastolic blood pressure, *EPAP* expiratory positive airway pressure, *HR* heart rate, *IPAP* inspiratory positive airway pressure, *IQR* interquartile range, *OI* oxygenation index, *RASS* Richmond Agitation Sedation Scale, *RR* respiratory rate, *SBP* systolic blood pressure, *SD* standard deviation

^a^Results are expressed as mean ± SD

^b^Results are expressed as n(%)

^c^Results are expressed as median (IQR)

### Outcomes

#### Failure of NIPPV and NIPPV duration

**Fig. 2:**
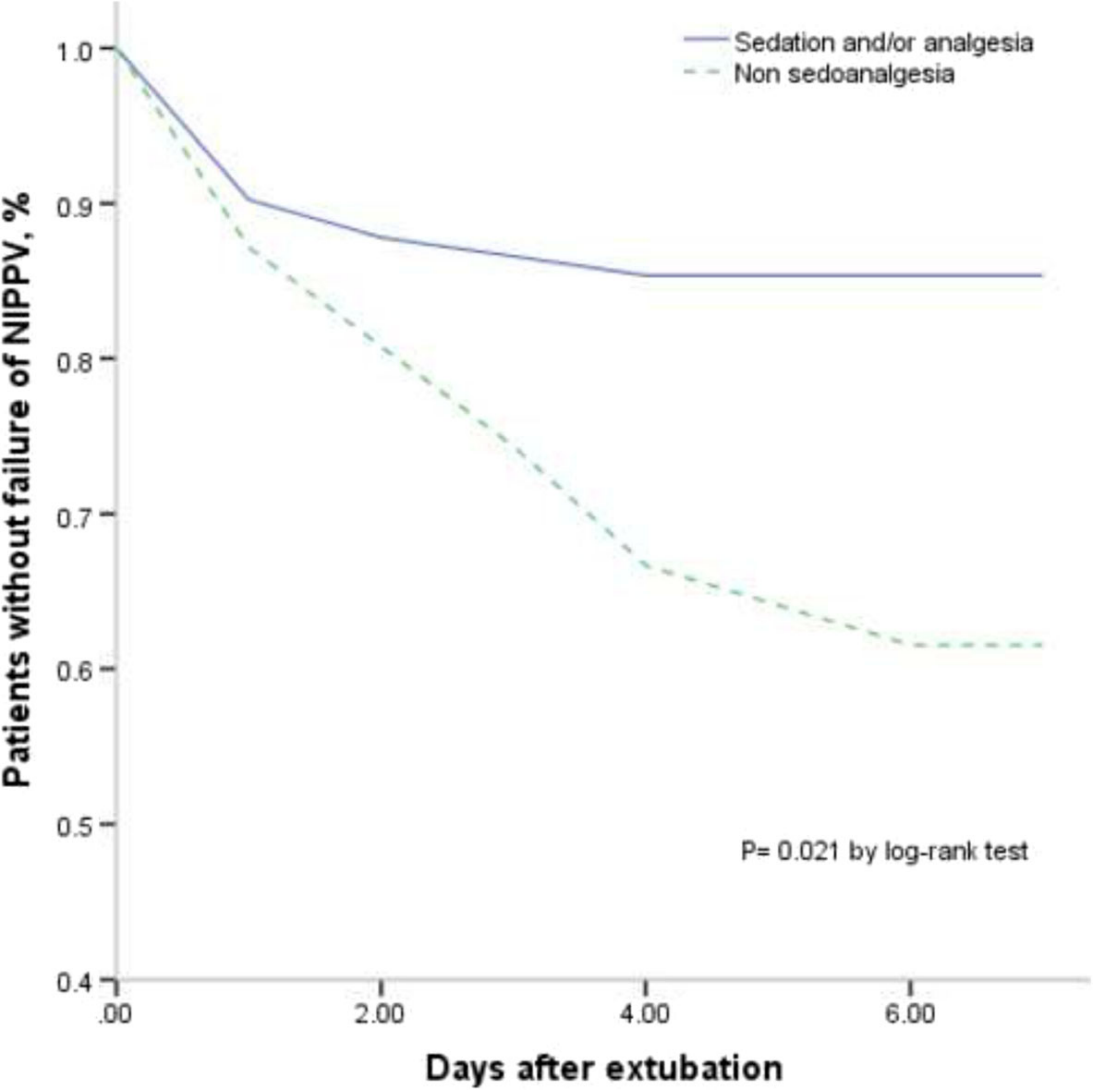
Kaplan-Meier curve of patients without failure of NIPPV after extubation. NIPPV noninvasive positive pressure ventilation

Overall, 21 of 80 patients (26%) failed NIPPV. (Table 2) The characteristics of patients with failed and successed NIPPV were presented in Table 2. No difference was found between two groups. In the unadjusted analysis, sedation and/or analgesia was significant associated with failure of NIPPV (29% vs. 59%, *P* = 0.015). After adjusting for sex before extubation (Table 3), we found that sedation and/or analgesia can reduce the rate of NIPPV failure (OR 0.29, 95% CI 0.10–0.86, *P* = 0.025). In the log-rank test, significant differences also existed in median time from extubation to failure of NIPPV (*P* = 0.021) (Fig. 2). Moreover, NIPPV duration was also shorter in the group of sedoanalgesia (46.5 vs. 70 h, *P* = 0.041)(Table ﻿4﻿).

**Table 2:** Comparison between patients with success of noninvasive positive pressure ventilation and patients with noninvasive positive pressure ventilation failure

	Failure NIPPV (*N* = 21)	Successful NIPPV (*N* = 59)	*P*
Sedoanalgesia^b^	6(29%)	35(59%)	0.015
Age^c^, years	65.00(53.00–78.00)	74.00(60.00–82.00)	0.148
Male^b^	11 (52%)	44 (75%)	0.098
APACHEII at ICU admission^a^	18.57 ± 5.31	18.61 ± 7.14	0.982
APACHEII before extubation^a^	10.86 ± 4.78	10.76 ± 4.58	0.936
NIPPV before mechanical ventilation^b^	7(33%)	15(25%)	0.486
Diagnosis			
Pneumonia^b^	13(62%)	36(61%)	0.943
AECOPD^b^	6(29%)	19(32%)	0.758
Other^b^	2(10%)	4(7%)	0.682
Interface			0.426
Oral-nasal mask^b^	18(86%)	54(92%)	
Nasal mask^b^	3(14%)	5(8%)	
Before extubation			
OI^a^	255.23 ± 61.49	241.54 ± 74.89	0.455
pH^a^	7.44 ± 0.02	7.43 ± 0.04	0.365
PaCO_2_^a,^ mmHg	46.54 ± 11.03	49.75 ± 10.82	0.249
Body temperature^a^	36.80 ± 0.45	36.83 ± 0.48	0.874
SBP^a﻿^,﻿ mmHg﻿﻿	137.29 ± 19.73	139.17 ± 21.10	0.722
DBP^a^, ﻿mmHg	71.81 ± 11.66	74.75 ± 11.97	0.334
RR^a^, ﻿bpm	21.43 ± 5.56	21.63 ± 5.60	0.889
HR^a^,﻿ bpm﻿	103.10 ± 15.39	103.68 ± 17.37	0.892
Arterial blood gases half an hour after NIPPV initiated			
OI^a^	210.00 ± 62.18	259.80 ± 273.49	0.413
pH^a^	7.42 ± 0.06	7.44 ± 0.04	0.101
PaCO_2_^a﻿^, ﻿mmHg	51.22 ± 13.61	50.27 ± 12.43	0.768
IPAPmax^a^, cmH_2_O,	12.81 ± 2.56	12.90 ± 2.40	0.887
EPAPmax^a^, cmH_2_O,	5.71 ± 1.27	5.42 ± 0.97	0.281
RASS			0.294
-2 to 0 point^b^	3(14%)	15(22%)	
1 to 2 point^b^	18(86%)	44(78%)	

*AECOPD* acute exacerbation of chronic obstructive pulmonary disease, *APACHE II* The Acute Physiologic and Chronic Health Evaluation, *DBP* diastolic blood pressure, *EPAP* expiratory positive airway pressure, *HR* heart rate, *ICU LOS* length APACHE II The Acute Physiologic and Chronic Health Evaluation, *DBP* diastolic blood pressure, *EPAP* expiratory positive airway pressure, HR heart rate, *ICU LOS* length of intensive care unit stay, *IPAP* inspiratory positive airway pressure, *IQR* interquartile range, *OI* oxygenation index, *RASS* Richmond Agitation Sedation Scale, *RR* respiratory rate, *SBP* systolic blood pressure, *SD* standard deviation

^a^Results are expressed as mean ± SD

^b^Results are expressed as n(%)

^c^Results are expressed as median (IQR)

**Table 3:** Effect of sedoanalgesia on clinical outcomes of patients with noninvasive positive pressure ventilation

	Crude		Adjusted	
	Odds ratio (95%CI)	*P* value	Odds ratio (95%CI)	*P* value
Faliure of NIPPV	0.27 (0.09 ~ 0.81)	0.015	0.29 (0.10 ~ 0.86)	0.025
Hospital mortality	0.16 (0.04 ~ 0.61)	0.004	0.14 (0.03 ~ 0.60)	0.008

*CI* confidence interval, *NIPPV* noninvasive positive pressure ventilation

**Table 4:** Results of patients according to sedonalgesia status during NIPPV

	Sedation and/or analgesia (N = 41)	Non sedoanalgesia (N = 39)	*P*
Delirium^a^	12 (29%)	7 (18%)	0.234
ICU LOS^b^, day	5.0 (3.0–9.0)	8.0 (6.0–15.0)	0.030
NIPPV duration^b^, hour	46.50(22.25 ~ 73.75)	70.00(40.00 ~ 120.00)	0.041
Failure of NIPPV^a^	6(15%)	15 (38%)	0.015
Mortality^a^	3 (7%)	13 (33%)	0.004

*ICU LOS* length of intensive care unit stay, *IQR* interquartile range, *SD* standard deviation

^a^Results are expressed as n(%)

^b^Results are expressed as median (IQR)

#### Hospital mortality

**Fig. 3:**
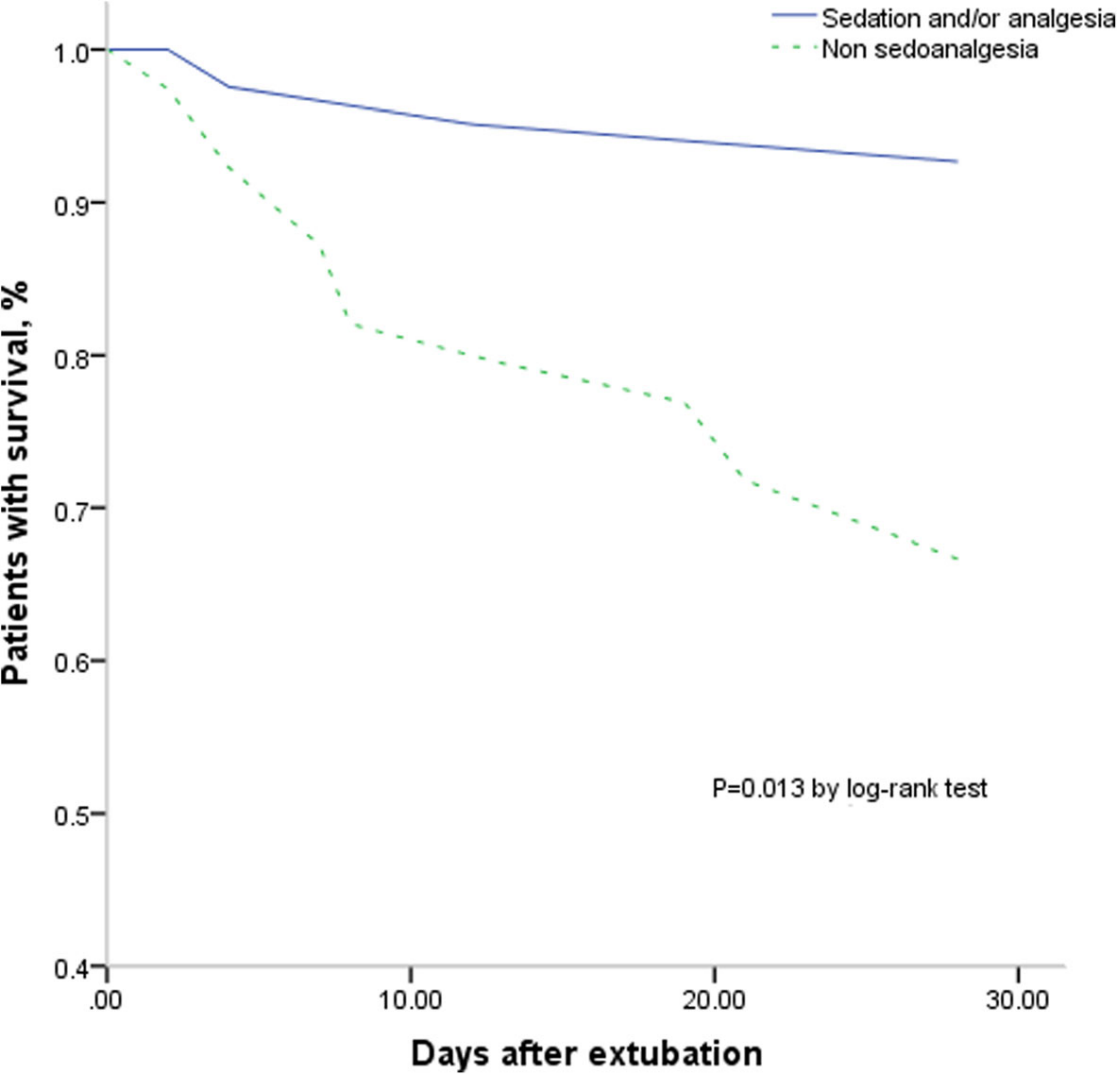
Kaplan-Meier curve of patients survived after extubation

Table 5 presented the characteristics of the patients survived and dead. No difference was found about the b﻿asic characteristics between the two groups. Hospital mortality was lower in patients who received sedation and/or analgesia compared with those who did not (19% vs. 59%, *P* = 0.004). After adjustment for PaCO_2_ before extubation and half an hour after NIPPV initiate, diagnosis and SBP, the difference remained significantly (Table 3): OR 0.14, 95% CI 0.03–0.60 (*P* = 0.008). In the log-rank test, significant differences also existed in median time from extubation to death (*P* = 0.013) (Fig. 3).

**Table 5:** Comparison between patients who were death and survival

	Death (*N* = 16)	Survival (*N* = 64)	*P*
sedoanalgesia^b^	3(19%)	38 (59%)	0.004
Age^c^, years	72.5(50.75 ~ 84)	71.5(59.75 ~ 81)	0.838
Male^b^	10 (63%)	45 (70%)	0.558
APACHEII at ICU admission^a^	19.63 ± 7.27	18.34 ± 6.56	0.496
APACHEII before extubation^a^	12.25 ± 5.71	10.42 ± 4.25	0.156
NIPPV before mechanical ventilation^b^	5(31%)	17(27%)	0.758
Diagnosis			
Pneumonia^b^	13(81%)	36(56%)	0.066
AECOPD^b^	1(6%)	24(38%)	0.016
Other^b^	2(13%)	4(6%)	0.396
Interface			0.657
Oral-nasal mask^b^	14(88%)	58(91%)	
Nasal mask^b^	2(12%)	6(9%)	
Before extubation			
OI^a^	248.54 ± 65.82	244.28 ± 73.31	0.833
pH^a^	7.44 ± 0.02	7.44 ± 0.04	0.588
PaCO_2_^a^, mmHg	43.68 ± 9.83	50.21 ± 10.83	0.031
Body temperature^a^	36.95 ± 0.50	36.79 ± 0.46	0.211
SBP^a^, mmHg	132.50 ± 12.03	140.22 ± 22.09	0.065
DBP^a^, mmHg	73.19 ± 8.83	74.17 ± 12.59	0.719
RR^a^, bpm	23.19 ± 6.96	21.17 ± 5.13	0.196
HR^a^, bpm	104.00 ± 18.86	103.41 ± 16.38	0.900
Arterial blood gases half an hour after NIPPV initiated			
OI^a^	225.90 ± 242.66	251.93 ± 264.81	0.698
pH^a^	7.43 ± 0.04	7.44 ± 0.05	0.458
PaCO_2_^a^, mmHg	44.86 ± 8.80	51.93 ± 13.15	0.045
IPAPmax^a^, cmH_2_O	12.44 ± 2.56	12.98 ± 2.40	0.423
EPAPmax^a^, cmH_2_O	5.38 ± 1.15	5.53 ± 1.04	0.600
RASS			0.284
-2 to 0 points^b^	2(13%)	16(25%)	
1 to 2 points^b^	14(87%)	48(75%)	

*AECOPD* acute exacerbation of chronic obstructive pulmonary disease, *APACHE II* The Acute Physiologic and Chronic Health Evaluation, *DBP* diastolic blood pressure, *EPAP* expiratory positive airway pressure, *HR* heart rate, *ICU LOS* length of intensive care unit stay, *IPAP* inspiratory positive airway pressure, *IQR* interquartile range, *OI* oxygenation index, *RASS* Richmond Agitation Sedation Scale, *RR* respiratory rate, *SBP* systolic blood pressure, *SD* standard deviation

^a^Results are expressed as mean ± SD

^b^Results are expressed as n(%)

^c^Results are expressed as median (IQR)

#### ICU LOS after extubation

ICU LOS was shorter in patients who received sedation and/or analgesia vs. those who did not receive drugs (5 vs. 8 days, *P* = 0.030).

Moreover, the rate of delirium of sedoanalgesia group was not different from the one of non-sedoanalgesia group (29% vs. 18%, *P* = 0.234).

## Discussion

In this retrospective study, patients with interface intolerance after extubation could benefit from sedation and/or analgesia during NIPPV treatment, resulting in the decrease of NIPPV failure rate, hospital mortality, and length of ICU stay after extubation. Our study showed that sedation and/or analgesia could reduce the rate of NIPPV failure and NIPPV duration. The mechanisms that explain the decrease of NIPPV failure rate and NIPPV duration have been reported as the followings. Firstly, the rate of delirium and NIPPV duration can be decreased when sedation and/or analgesia treatment applied because the treatment can relieve anxiety and stimulate sleep [15–17]. In addition, physiology responses to stress such as hypertension and tachycardia can also be modulated, which might facilitate NIPPV [18] and improve the respiratory status of patients. Secondly, sedation and/or analgesia could abate the uncomfortable feelings about NIPPV especially in terms of the interface intolerance, resulting in improved synchronizing between patients and ventilator, which could promote gas exchange [11, 12]. A study about 36 patients showed that patients who complained of discomfort and asked for interruption of NIPPV session had lower rate of failure when received remifentanil-based sedation protocol [19]. Moreover, although the potential effects of sedation and/or analgesia on depressing respiration and hypoxic drive were worried by many clinicians, physiological studies on the impacts of sedation and/or analgesia on ventilator response demonstrated that a continuous infusion of sedation and/or analgesia will not have significant influence on respiratory drive, respiratory pattern, minute volume, and blood gases [20–22]. In our study, we found that sedation and/or analgesia would not influence the oxygenation and that the OI of patients in the sedation and/or analgesia remained unchanged. On the contrary, the extraction of CO_2_ in patients can be facilitated by conducting sedoanalgesia, showed by the lower PaCO_2_ after the administration of the drug (51.33 ± 13.49 vs. 48.53 ± 13.54 mmHg, *P =* 0.019). Also, the study of Clouzeau et al. found a significant increase of oxygenation index in 10 acute respiratory failure (ARF) patients (from 167 ± 68 to 195 ± 68; *P* < 0.05) to verify the safety of sedation and analgesia [23]. A result from another study about 13 patients also support this point of view (PaCO_2_ decrease from 57.8 ± 15.3 to 49 ± 9.8 mmHg, *P* < 0.05) [11].

However, some other studies showed opposite results. They found that the use of sedation and/or analgesia may be related to the failure of NIPPV [24]. The different result might be caused by the discrepancy between patients enrolled in our study and theirs. In our study, only the patients with interface intolerance according to the records were enrolled in, while in other studies, the premise is uncertain. Therefore, the exact clinical aim of the sedation and/or analgesia might not be comforting patients or facilitating the NIPPV in those studies, but patients may suffer from the adverse events of sedation and/or analgesia, such as hypotension, arrhythmias, respiratory acidosis, and infections from contaminated vials or tubing [25]. In our study, NIPPV was applied after extubation, when patients received respiratory support mainly due to low ability of airway protection and cough strength [26]. On the contrary, when NIPPV was used as first line therapy in the other studies, the initial disease,which often had been released in the patients after extubation, was the primary problem of ARF. Further more, in our study, the RASS score of the patients enrolled in our study was controlled between −2 to 2, while the RASS score of a big proportion of patients in other studies were lower than −2. As we all know, excess sedation will bring numerous complications such as delirium, and result in increasing mortality [27].

We also found the management of sedation and/or analgesia could reduce the hospital mortality and ICU LOS after extubation. This may mainly due to the lower rate of NIPPV failure in the patients received sedation and/or analgesia [28–30]. Studies showed that a lot of the adverse events caused by the reintubation were related to invasive mechanical ventilation that would multiple the rate of mortality such as ventilator associated pneumonia (VAP), ventilator-associated lung injury and barotrauma [31–33].

A recent study showed that VAP would affect approximately 10% of the ventilated patients [34]. It was a risk factor for ICU mortality (OR 2.20; 95%CI 1.91–2.54) [35] and prolonged the length of hospital stay compared with patients without VAP (22.7 ± 2.9 vs. 16.8 ± 2.9; *P* < 0.0006) [36]. Moreover, Studies showed that the rate of barotrauma induced by invasive mechanical ventilation can be up to 50% and the mortality of patients with barotrauma also increased (51.4 vs 39.2%; *P* = 0.04) as well as the ICU LOS (14 ± 13.6 vs. 10.9 ± 11.4; *P* = 0.04) [37].

Moreover, our study also showed that sedoanalgesia could better improve the prognosis of patients with hypercapnia than the ones only with hypoxemia. This might because that NIPPV plays different role in different occasions and different diseases [38]. For example, the advantages of NIPPV used in patients with AECOPD has been recognized already, but whether NIPPV can improve the clinical outcomes in patients with asthma remains debated [39, 40].

Our study has several limitations. The small sample size of this study did not allow us to divide patients into sedation, analgesia, sedation and/or analgesia, and non sedoanalgesia groups. Moreover, in the clinical practice, it is difficult to distinguish the potential role of the two kinds of drugs because both of the sedative and analgesia drugs had the underlying function of respiratory inhibition and can have significant impact on moderating the intolerance. Moreover, because of shortcomings of retrospective study, we could not unify the criterion of the drug dose and the duration of administration. Also, other potential factors contribute to the clinical outcomes such as the ability of airway protection and cough strength also were unable to measure.

## Conclusion

The use of sedation and/or analgesia could improve the survival rate among patients with interface intolerance after extubation and decrease the rate of NIPPV failure and ICU LOS, especially in patients with hypercapnia. Therefore, under the 24 h monitoring in the ICU, sedation and/analgesia is safe and effective in patients with interface intolerance after extubation. However, more large randomized controlled trials are still needed to verify the result.

## Additional files

Additional file 1: Weaning protocol and standard of reintubation. (DOC 19 kb)

Additional file 2: Inclusion criteria for patients used NIPPV directly after extubation. (DOC 19 kb)

## Abbreviations

APACHE: The Acute Physiologic and Chronic Health Evaluation
CI: Confidence interval
FiO_2_: Fraction of inspired oxygen
GOLD: The Global Initiative for Chronic Obstructive Lung Disease
ICU: Intensive care unit
LOS: Length of stay
MV: Mechanical ventilation
NIPPV: Noninvasive positive pressure ventilation
PaCO_2_: Arterial partial pressure of carbon dioxide
PaO_2_: Arterial partial pressure of oxygen
RASS: Richmond agitation sedation scale
SD: Standard derivation
