# Supplementary material for: The effect of sedation and/or analgesia as rescue treatment during noninvasive positive pressure ventilation in the patients with Interface intolerance after Extubation
Source: BMC Pulm Med. 2017 Sep 15;17:125. doi: 10.1186/s12890-017-0469-4 (PMC5602861; doi:10.1186/s12890-017-0469-4)
Supplement: Supplementary file 1 — Weaning protocol and standard of reintubation. (DOC 19 kb) [file 12890_2017_469_MOESM1_ESM.doc]

1. **Weaning Protocol**

The weaning protocol included daily screening for weaning readiness according to the following criteria:

1)Recovery from the precipitating illness;

2)Respiratory criteria(PaO2:FiO2[partial pressure of oxygen, arterial:fraction of inspired oxygen] ratio >150 with FiO2 ≦0.4，PEEP <8cmH20， and arterial pH>7.35);

3)Clinical criteria(absence of electrocardiographic signs of myocardial ischemia, no vasoactive drugs, or only low doses of dopamine[<5ug/kg/min],heart rate <140/min,hemoglobin >8g/dL,temperature <38^。^C,no need for sedatives, presence of respiratory stimulus, and appropriate spontaneous cough).

4)Patients fulfilling these criteria underwent a spontaneous breathing trial with pressure suppor for 30 to 120 minutes.

5)Patients who tolerated the spontaneous breathing trial were reconnected with the previous ventilator settings for rest and clinical evaluation of airway patency, respiratory secretions, and upperairway obstruction.

Reference:

1.Boles JM, Bion J, Connors A, Herridge M, Marsh B, Melot C, Pearl R, Silverman H, Stanchina M, Vieillard-Baron A, Welte T. Weaning from mechanical ventilation. Eur Respir J. 2007 May;29(5):1033-56.

**2. Standard of reintubation**

patients were reintubated for persistent postextubation respiratory failure if they met at least one of the following criteria:

1) Lack of improvement in pH or in the partial pressure of carbon dioxide or fall in GCS score >2 points.

2) Lack of improvement in signs suggestive of respiratory-muscle fatigue or worsening including the appearance of unequivocal signs of respiratory-muscle fatigue, such as maintained active contraction of the expiratory muscles, asynchronous motion of the rib cage and abdomen, respiratory alternans, or active contraction of the sternocleidomastoid.

3) Hypotension, with a systolic blood pressure below 90 mm Hg for more than 30 minutes despite adequate volume challenge, use of vasopressors, or both.

4) Copious secretions that could not be adequately cleared or that were associated with acidosis, hypoxemia, and changes in mental status or persistent or worsening signs of respiratory-muscle fatigue.

5) Decrease to SpO2 <85% despite the use of a high FiO2 (>.5).

Patients fulfilling these criteria were reintubated, but the final decision to reintubate was made by the treating physician or evaluated by a consensus committee excluding the investigators. The single most relevant reason for reintubation from the list was recorded. If two or more criteria were present, the reason for reintubation was assigned in the following order of preference: presence of copious secretions, respiratory acidosis, hypoxemia, signs of respiratory-muscle fatigue, and hypotension for selection of cause of reintubation.
