# Supplementary material for: The effect of sedation and/or analgesia as rescue treatment during noninvasive positive pressure ventilation in the patients with Interface intolerance after Extubation
Source: BMC Pulm Med. 2017 Sep 15;17:125. doi: 10.1186/s12890-017-0469-4 (PMC5602861; doi:10.1186/s12890-017-0469-4)
Supplement: Supplementary file 2 — Inclusion criteria for patients used NIPPV directly after extubation. (DOC 19 kb) [file 12890_2017_469_MOESM2_ESM.doc]

1. **Inclusion criteria for patients used NIPPV directly after extubation**

Patients fullfilling at least 1 of the following criteria were given NIPPV directly after extubation:

1)Age older than 65 years;

2)Heart failure as the primary indication for mechanical ventilation;

3)Moderate to severe chronic obstructive pulmonary disease;

4)An Acute Physiology and Chronic Health Evaluation II(APACHE II) score higher than 12 on extubation day;

5)Body mass index of more than 30(calculated as weight in kilograms divided by height in meters squared);

6)Airway patency problems, including high risk of developing laryngeal edema;

7)Inability to deal with respiratory secretions(inadequate cough reflex or suctioning>2 times within 8 hours before extubation);

8)Difficult or prolonged weaning, in brief, a patient failing the first attempt at disconnection from mechanical ventilation;

9)2 or more comorbidities;

10)Mechanical ventilation for more than 7 days.

**Reference:**

1.Nava S, Gregoretti C, Fanfulla F, Squadrone E, Grassi M, Carlucci A, Beltrame F, Navalesi P. Noninvasive ventilation to prevent respiratory failure after extubation in high-risk patients. Crit Care Med. 2005 Nov;33(11):2465-70.

2.Ferrer M, Valencia M, Nicolas JM, Bernadich O, Badia JR, Torres A. Early noninvasive ventilation averts extubation failure in patients at risk: a randomized trial. Am J Respir Crit Care Med. 2006 Jan 15;173(2):164-70.

3.El-Solh AA, Aquilina A, Pineda L, Dhanvantri V, Grant B, Bouquin P. Noninvasive ventilation for prevention of post-extubation respiratory failure in obese patients. Eur Respir J. 2006 Sep;28(3):588-95.

4.Thille AW, Harrois A, Schortgen F, Brun-Buisson C, Brochard L. Outcomes of extubation failure in medical intensive care unit patients. Crit Care Med. 2011 Dec;39(12):2612-8.

**5.**Vallverdú I, Calaf N, Subirana M, Net A, Benito S, Mancebo J. Clinical characteristics, respiratory functional parameters, and outcome of a two-hour T-piece trial in patients weaning from mechanical ventilation. Am J Respir Crit Care Med. 1998 Dec;158(6):1855-62.
